# Enhanced identification of ventricular tachycardia isthmus within a scar using Ripple map and Octaray catheter

**DOI:** 10.1016/j.ipej.2024.03.001

**Published:** 2024-03-08

**Authors:** Masato Okada, Koji Tanaka, Nobuaki Tanaka

**Affiliations:** Cardiovascular Center, Sakurabashi Watanabe Hospital, Osaka, Japan

**Keywords:** Catheter ablation, Critical isthmus, Electroanatomic mapping, Ventricular tachycardia

## Abstract

• Low-amplitude complex EGMs are often observed in the VT isthmus.
• Misannotation of local EGMs can lead to incorrect depiction of VT circuits.
• Ripple map with Octaray catheter can enhance VT isthmus identification.

## Descriptions

**Fig. 1:**
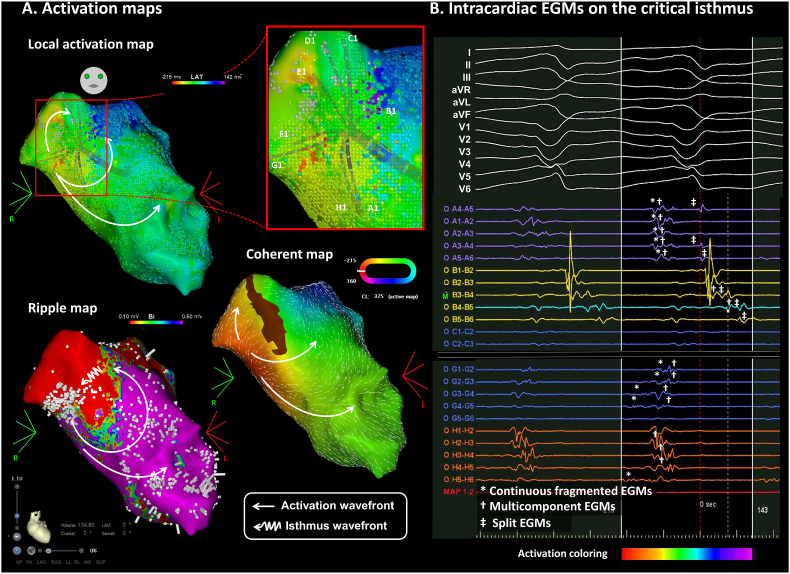
**A.** Electroanatomic mapping using the CARTO 3 system and Octaray catheter during ventricular tachycardia. Ripple map was projected over the voltage map, and bars were set to display electrograms above 0.03 mV and were clipped above 0.5 mV for easier visualization. **B.** Intracardiac electrograms recorded by the Octray catheter during ventricular tachycardia.

An 80-year-old man with hypertrophic cardiomyopathy was referred for catheter ablation of recurrent ventricular tachycardia (VT). Under general anesthesia, substrate and activation maps were created using an Octaray catheter and CARTO 3 system (Biosense Webster, Irvine, CA). At baseline, scar and a low voltage area (LVA) were observed on the basal anterior septum of the left ventricle. During the VT, the local activation map and Coherent map revealed a centrifugal spread from the bottom of the LVA. However, the Ripple map revealed a clear VT circuit with its isthmus within the scar (<0.5 mV) (Fig. 1A). An application of radiofrequency energy at the isthmus successfully terminated the VT, and it was never induced thereafter.

Low-amplitude complex electrograms are often observed in the critical isthmus of VTs (Fig. 1B) [1]. Thus, relying solely on fixed-point annotations of these electrograms can lead to inaccuracies in identifying the VT circuit. In this case, the automatic dV/dt annotation algorithms overlooked the tiny diastolic potentials, focusing instead on the larger outer loop potentials. The Ripple map, which aligns all recorded electrograms at each point, can display all the information on the complex fractionated electrograms without mis-annotation [2]. The dynamic images allow us to distinguish whether the tiny fractionated potentials are activated with nearby normal myocardium during the systolic phase (far-field electrograms) or solely during the diastolic phase (near-field electrograms). The Octaray catheter can further enhance the Ripple map utility by recording a larger number of samples with more accurate unipolar electrograms [3]. When using the 3-dimensional mapping system for complex VTs, operators should recognize how the tiny fractionated continuous electrograms are handled with each activation map.

## Patient consent statement

Informed consent was obtained from the patient to publish this case report and accompanying images.

## Funding

None.

## Declaration of generative AI and AI-assisted technologies in the writing process

None.

## Declaration of competing interest

The authors declare that they have no known competing financial interests or personal relationships that could have appeared to influence the work reported in this paper.
